# Geometric morphometric wing analysis as a tool to discriminate female mosquitoes from different suburban areas of Chiang Mai province, Thailand

**DOI:** 10.1371/journal.pone.0260333

**Published:** 2021-11-29

**Authors:** Danita Champakaew, Anuluck Junkum, Narin Sontigun, Sangob Sanit, Kwankamol Limsopatham, Atiporn Saeung, Pradya Somboon, Benjawan Pitasawat

**Affiliations:** 1 School of Public Health, Walailak University, Nakhon Si Thammarat, Thailand and Excellent Center for Dengue and Community Public Health (EC for DACH), Nakhon Si Thammarat, Thailand; 2 Department of Parasitology, Center of Insect Vector Study, Faculty of Medicine, Chiang Mai University, Chiang Mai, Thailand; 3 Akkhraratchakumari Veterinary College, Walailak University, Nakhon Si Thammarat, Thailand; Beni Suef University Faculty of Veterinary Medicine, EGYPT

## Abstract

Mosquitoes are hematophagous insects that transmit parasites and pathogens with devastating effects on humans, particularly in subtropical regions. Different mosquito species display various behaviors, breeding sites, and geographic distribution; however, they can be difficult to distinguish in the field due to morphological similarities between species and damage caused during trapping and transportation. Vector control methods for controlling mosquito-borne disease epidemics require an understanding of which vector species are present in the area as well as the epidemiological patterns of disease transmission. Although molecular techniques can accurately distinguish between mosquito species, they are costly and laborious, making them unsuitable for extensive use in the field. Thus, alternative techniques are required. Geometric morphometrics (GM) is a rapid and inexpensive technique that can be used to analyze the size, shape, and shape variation of individuals based on a range of traits. Here, we used GM to analyze the wings of 1,040 female mosquitoes from 12 different species in Thailand. The right wing of each specimen was removed, imaged microscopically, and digitized using 17 landmarks. Wing shape variation among genera and species was analyzed using canonical variate analysis (CVA), while discriminant function analysis was used to cross-validate classification reliability based on Mahalanobis distances. Phenetic relationships were constructed to illustrate the discrimination patterns for genera and species. CVA of the morphological variation among *Aedes*, *Anopheles*, *Armigeres*, *Culex*, and *Mansonia* mosquito genera revealed five clusters. In particular, we demonstrated a high percentage of correctly-distinguished samples among *Aedes* (97.48%), *Armigeres* (96.15%), *Culex* (90.07%), and *Mansonia* (91.67%), but not *Anopheles* (64.54%). Together, these findings suggest that wing landmark-based GM analysis is an efficient method for identifying mosquito species, particularly among the *Aedes*, *Armigeres*, *Culex*, and *Mansonia* genera.

## Introduction

Mosquitoes are hematophagous insects that are considered to be one of the most dangerous vectors in the world due to their potential to transmit parasites and pathogens responsible for serious diseases, including malaria, filariasis, yellow fever, dengue, and Japanese encephalitis [1]. Indeed, over 1 billion cases and 1 million deaths due to mosquito-borne diseases are reported annually [2], with mosquitoes becoming an increasing problem in tropical and subtropical regions [3]. Although more than 3,000 species of mosquito exist worldwide, the main vectors of clinical importance are *Anopheles* spp. (malaria, lymphatic filariasis, and Japanese encephalitis), *Culex* spp. (lymphatic filariasis, Japanese encephalitis), *Aedes* spp. (dengue/dengue hemorrhagic fever, yellow fever, lymphatic filariasis), and *Mansonia* spp. (lymphatic filariasis) [4, 5]. In Thailand, there are over 400 species of mosquito and the major vectors of mosquito-borne diseases are *Anopheles* spp. (*An*. *dirus* and *An*. *minimus*), *Mansonia* spp. (*Mn*. *annulata* and *Mn*. *annulifera*), *Culex* spp. (*Cx*. *tritaeniorhynchus*), and *Aedes* spp. (*Ae*. *aegypti* and *Ae*. *albopictus)* [6].

Vector control methods are an important strategy for controlling mosquito-borne disease epidemics [7]; however, their success relies on a good understanding of the biology and geographic distribution of mosquito vectors [8]. Since different mosquito species have different characteristics, such as behavior, breeding site, and geographic distribution, their accurate identification is of great importance for medical entomology [9]. The most common method of species identification relies on morphological taxonomic keys and is a laborious process that requires intensive training. Unfortunately, it can be difficult to identify mosquito vectors in the field based on morphological features due to the prevalence of cryptic, sibling, or isomorphic species with similar genetics and morphologies. Although some mosquito species are easily distinguished when in good condition, morphological identification is often hampered by damage to the external characteristics of field specimens during trapping and transportation [10] or through their preservation in ethanol [11, 12]. Consequently, several molecular or phenotypic tools have been developed for the identification of problematic species. Although molecular techniques can accurately distinguish mosquito species, they are very costly and labor intensive, making them unsuitable for routine use in the field where many samples are collected. Therefore, novel alternatives to classical morphology or DNA identification are required to identify mosquito vectors in the field.

Morphometric methods, such as geometric morphometrics (GM), are a rapid, inexpensive, and valuable tool for analyzing the biological size, shape, and shape variation of individuals based on various traits [13]. GM has been applied extensively in a number of fields, including entomology, where it has been used to analyze blow flies, mosquitoes, bees, and Triatominae eggs [14]. In addition, several recent studies have used GM to classify species and examine variation among clinically important mosquitoes that are morphologically similar or are sibling species [15–17]. Currently, GM based on the geometry of wing features (landmark-based) is largely used in medical entomology to reliably distinguish between closely related species [18, 24]. In particular, wing landmark-based GM has been successfully used to identify three *Stomoxys* fly species that are difficult to separate using external morphological characteristics, with a correct classification rate of 76–100% [19]. Wing landmark-based GM has also been used to distinguish 12 medically and forensically important blow fly species in Thailand at both the genus and species levels [18]. Moreover, GM analysis has been used to identify clinically important mosquito species, sibling species, or cryptic species based on adult female wing morphometry alone [15, 20–24]. In this study, we aimed to determine whether the wing landmark-based GM analysis can be used as an identification tool for clinically important mosquito genera/species in Thailand.

## Materials and methods

### Mosquito samples and identification

A total of 1,040 female mosquitoes were collected from field and laboratory colonies for use in this study (Table 1). Free-mating laboratory colonies of mosquito vectors from the *Aedes*, *Anopheles*, and *Culex* genera were reared and maintained continually in the insectary of the Department of Parasitology, Faculty of Medicine, Chiang Mai University, Chiang Mai province, Thailand. The colonies were housed without exposure to any pathogens or insecticides at a constant temperature of 27±2°C and 70–80% relative humidity under a 12:12 h light/dark photoperiod. Laboratory colonies of *Ae*. *aegypti*, *An*. *cracens* (formerly *An*. *dirus* B), *An*. *dirus*, *An*. *minimus* sensu stricto (formerly *An*. *minimus* A), and *Cx*. *quinquefasciatus* were reared and maintained in an insectary for several generations. *Ae*. *aegypti* (Muang Chiang Mai-susceptible: MCM-S), *An*. *cracens*, *An*. *dirus*, *An*. *minimus*, and *Cx*. *quinquefasciatus* (National Institute of Health of Thailand: NIH strain) originated from field larvae collected originally in Chiang Mai province, Muang Chiang Mai district in 1995, the Armed Forces Research Institute of Medical Sciences (AFRIMS, Bangkok, Thailand), the Vector Borne Disease Section, Office of Disease Prevention and Control No. 10 (Chiang Mai, Thailand), and the National Institute of Health, Ministry of Public Health (Nonthaburi province, Thailand), respectively. Mass rearing was conducted according to previously described procedures [25], with slight modifications. Rearing trays containing aquatic stage mosquitoes were covered tightly at all times with a nylon screen in order to ensure that all colonies were strictly isolated from each other. Adults were fed continually with 10% sucrose and 10% v/v multivitamin syrup using soaked cotton pads. Female mosquitoes were collected using a mount aspirator, frozen at -20°C for 10 min, and stored in 80% ethanol.

**Table 1:** Mosquito specimens used in this study.

Mosquito species	Species code	Collection site		GPS reference		Total no. of female specimens	
		Subdistrict	Province	Latitude	Longitude		
*Aedes vexans*	Aeve	Maehia	Muang Chiang Mai	18° 44’ 15.9" N	98° 56’ 49" E	13	**30**
		Sri Phum		18° 47’ 28.8" N	98° 58’ 19.5" E	5	
		Sunpesua		18° 83’ 26" N	09° 00’ 15" E	12	
*Aedes aegypti*	Aeae	Maehia	Muang Chiang Mai	18° 44’ 15.9" N	98° 56’ 49" E	4	**56**
		Laboratory strain		n/a		52	
*Aedes albopictus*	Aeal	Maehia	Muang Chiang Mai	18° 44’ 15.9" N	98° 56’ 49" E	3	**33**
		Sunpesua		18° 83’ 26" N	09° 00’ 15" E	30	
*Anopheles dirus*	Andi	Laboratory strain		n/a		**36**	
*Anopheles cracens*	Ancr	Laboratory strain		n/a		**56**	
*Anopheles minimus*	Anmi	Laboratory strain		n/a		**49**	
*Armigeres subalbatus*	Arsu	Maehia	Muang Chiang Mai	18° 44’ 15.9" N	98° 56’ 49" E	21	**182**
		Sunpesua		18° 83’ 26" N	09° 00’ 15" E	161	
*Culex gelidus*	Cxge	Maehia	Muang Chiang Mai	18° 44’ 15.9" N	98° 56’ 49" E	4	**37**
		Sri Phum		18° 47’ 28.8" N	98° 58’ 19.5" E	13	
		Sunpesua		18° 83’ 26" N	09° 00’ 15" E	20	
*Culex vishnui*	Cxvi	Maehia	Muang Chiang Mai	18° 44’ 15.9" N	98° 56’ 49" E	21	**344**
		Sri Phum		18° 47’ 28.8" N	98° 58’ 19.5" E	10	
		Sunpesua		18° 83’ 26" N	09° 00’ 15" E	313	
*Culex quinquefasciatus*	Cxqu	Maehia	Muang Chiang Mai	18° 44’ 15.9" N	98° 56’ 49" E	9	**193**
		Sri Phum		18° 47’ 28.8" N	98° 58’ 19.5" E	34	
		Sunpesua		18° 83’ 26" N	09° 00’ 15" E	90	
		Laboratory strain		n/a		60	
*Mansonia indiana*	Mnin	Maehia	Muang Chiang Mai	18° 44’ 15.9" N	98° 56’ 49" E	2	**11**
		Sunpesua		18° 83’ 26" N	09° 00’ 15" E	9	
*Mansonia uniformis*	Mnun	Sunpesua	Muang Chiang Mai	18° 83’ 26" N	09° 00’ 15" E	**13**	
						**Total 1,040**	

**Fig 1:**
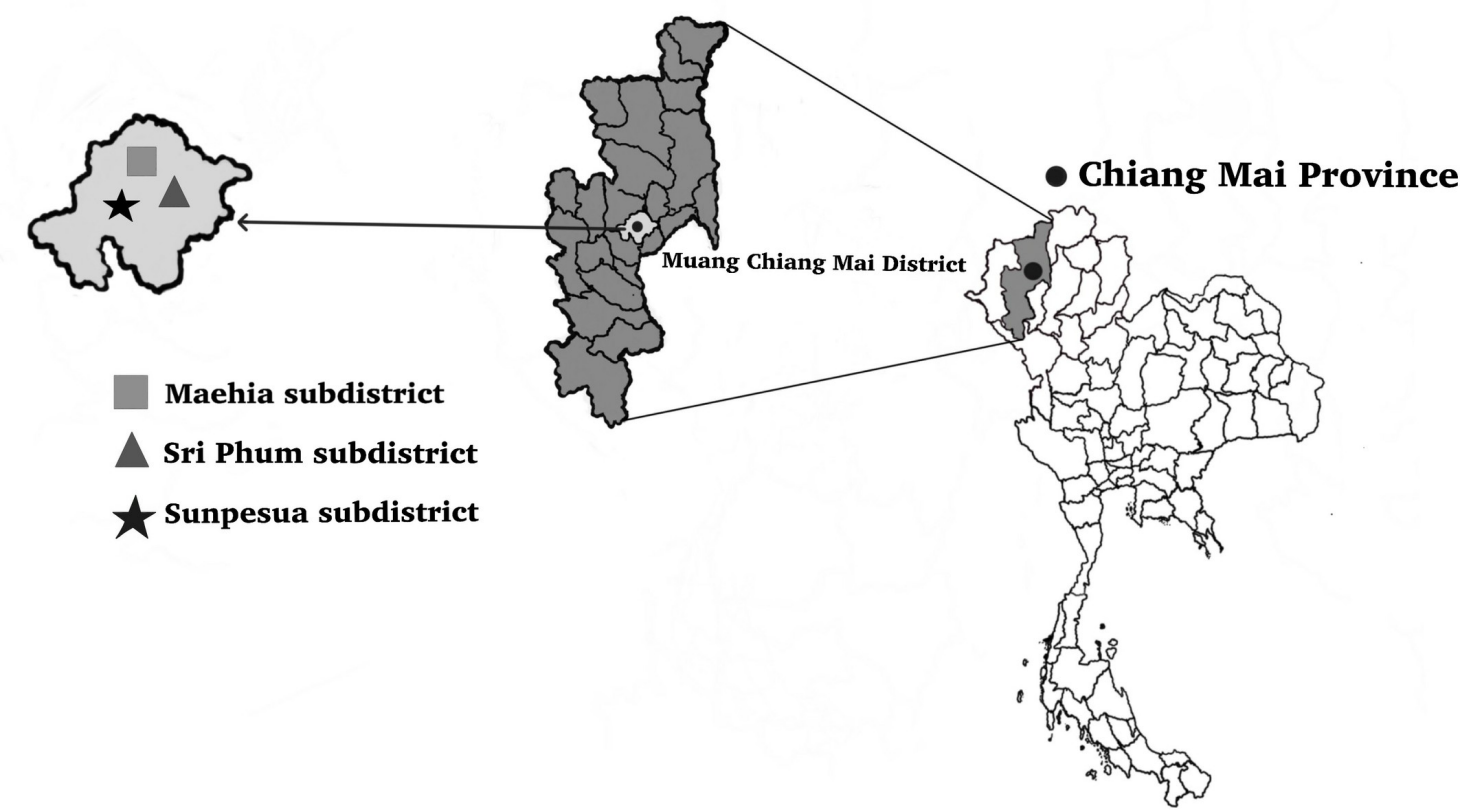
Map of Thailand showing the collection sites of adult mosquitoes used for wing morphometric analysis. Mosquitoes were collected from three subdistricts (Maehia, Sri Phum, and Sunpesua) in the Muang district of Chiang Mai province, northern Thailand.

Based on previous research [26–28], adult mosquitoes were collected from natural populations between 18:00 and 22:00 h using a modified human bait trap at suburban sites in three locations in Muang district, Chiang Mai province: Sunpesua, Maehia, and Sri Phum subdistrict. Field trials were conducted after receiving permission from the possessor of the private land. Each human volunteer was covered with a long-sleeved jacket with hood, shoes with socks, gloves, and long pants rolled up to the knee (exposed area: the lower leg). Mosquitoes landing on the exposed area of volunteers were mouth aspirated by proficient collectors before the natural mosquitoes could imbibe any blood. The captured insects were kept in paper cups for counting and identification. A total of 787 adult female mosquitoes from five genera, (*Aedes*, *Anopheles*, *Armigeres*, *Culex*, and *Mansonia*) were counted under a stereomicroscope and their species were identified using previously reported taxonomic keys [29] before they were stored in 80% ethanol (Table 1, Fig 1). This project was approved and conducted according to protocol PAR-2556-01588 of the Research Ethics Committee, Faculty of Medicine, Chiang Mai University.

### GM sample preparation

The right wing was dissected from the thorax of each female mosquito using an insect needle under a stereomicroscope (Olympus SZX7, Germany). The wings were then mixed with normal saline solution (NSS) in an Eppendorf tube using a vortexer (VORTEX-GENIE2, Scientific Industries, Inc., New York, United States) at shake level 3 for 3–5 min to remove the wing scales. Next, each wing was placed on a glass slide in one drop of distilled water and the remaining scales were removed using a small round paintbrush (No. 0).

### Image manipulation and data acquisition

**Fig 2:**
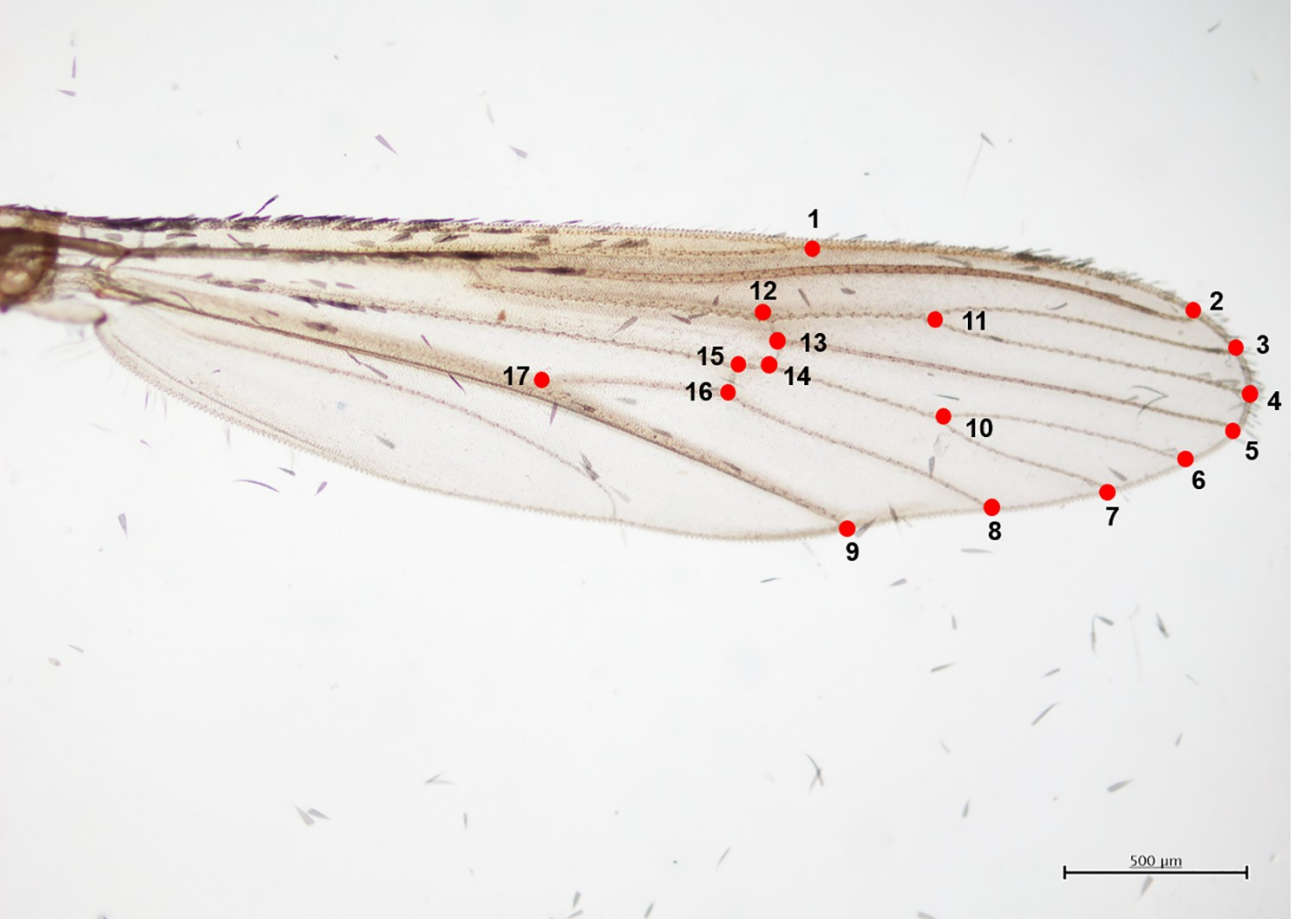
Representative image of wing landmark pattern. Right wing of female *Ae*. *aegypti* showing the 17 plotted landmarks based on Dujardin et al. (2017).

The wing was placed onto a glass microscope slide and photographed using a digital camera (Olympus DP22) connected to a light microscope under 4× magnification. A 500-μm scale bar was embedded into each image and kept in the same folder. Tps files were built from the images using TpsUtil32 v.1.78 software [30] to reduce liable marking (LM) bias when digitizing landmark locations. Seventeen selected landmarks [31–33] (Fig 2) were digitized using TpsDig2 v.2.31 software [34]. The LM of the venation pattern of each wing was digitized in duplicate to reduce measurement error by the same handler [35].

### GM analysis

The duplicate.tps files produced by digitizing wing landmark locations were used to measure the isometric estimator known as centroid size (CS), which is defined as the square root of the sum of the squared distances between the center of the LM or centroid configuration [36]. All samples were analyzed using MorphoJ software v.1.07a [37] and aligned and superimposed using the “Procrustes Fit” function to remove variation due to differences in scale, position, and orientation of the coordinates. The CS and Procrustes coordinates obtained from the landmark data were averaged for each specimen prior to further statistical analysis.

### Wing shape variation

To assess the effect of wing size on wing shape (allometry), the regression of the Procrustes coordinates (dependent variable) against CS (independent variable) was analyzed using a permutation test with 10,000 randomizations. The variations in wing shape between five genera/12 species of mosquitoes were determined using CVA, and the reliability of classification within each genus/species was confirmed using discriminant function analysis (DFA), a cross-validation test based on Mahalanobis distances. Additionally, each sample was reclassified according to the similarity of its wings to the average wing shape of all species using a pairwise cross-validated reclassification test based on Mahalanobis distances. All the analyses were conducted in MorphoJ software v.1.07, and a permutation test with 10,000 replications was used.

### Phenetic wing morphology relationships

A Neighbor-Joining (NJ) analysis was constructed with 1,000 bootstrap replicates based on Mahalanobis distances obtained through pairwise comparison of analyzed species via CVA using PAST software v.4.03 (https://past.en.lo4d.com/windows) to illustrate the phenetic relationships between the wing data of 12 mosquito species.

## Results

### Wing shape variation

**Fig 3:**
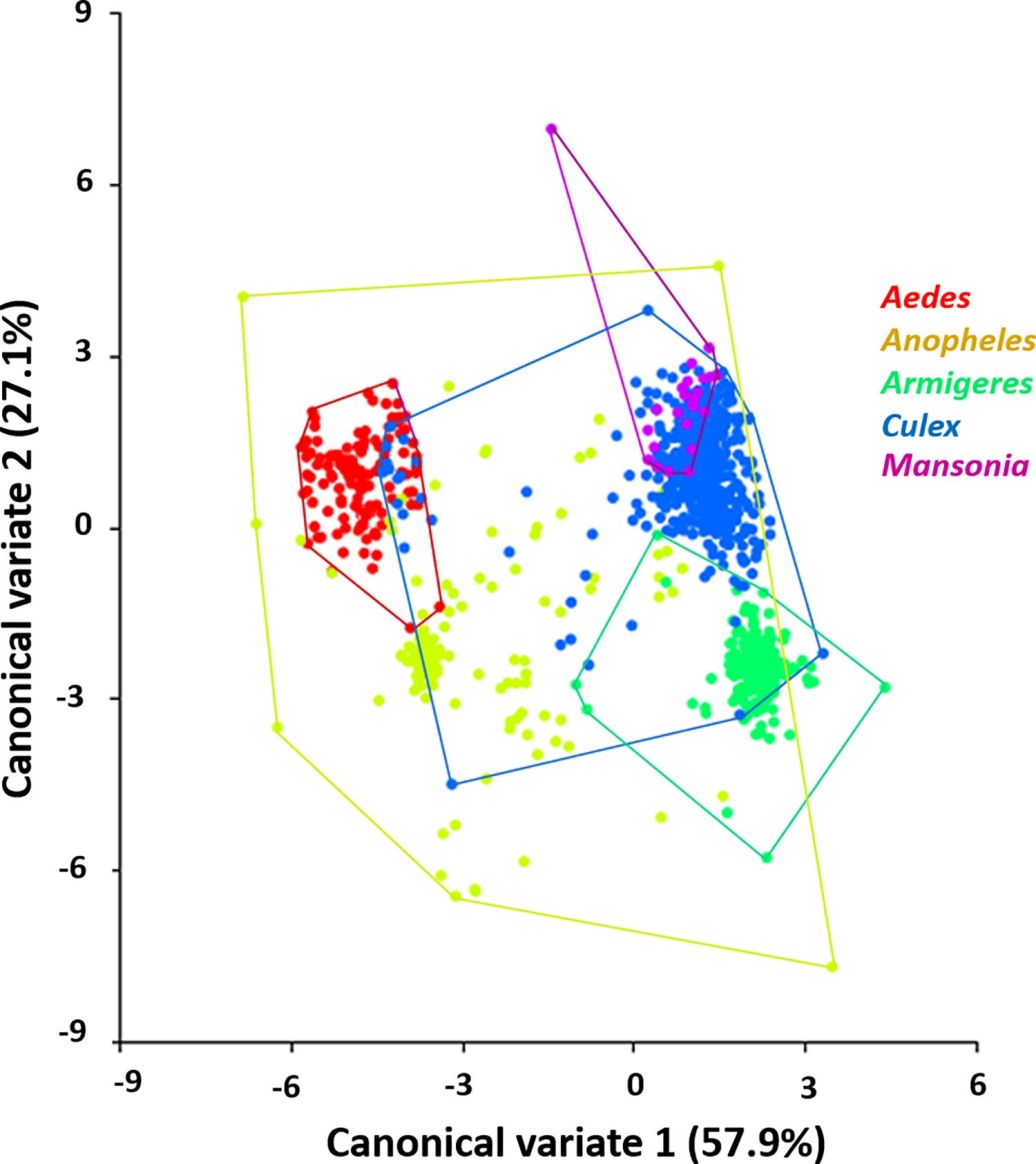
Scatter plot showing wing shape variation among five mosquito genera. Wing shape variation in *Aedes*, *Anopheles*, *Armigeres*, *Culex*, and *Mansonia* mosquitoes is shown along the first two canonical variate (CV1 and CV2) axes with 90% confidence ellipses.

**Fig 4:**
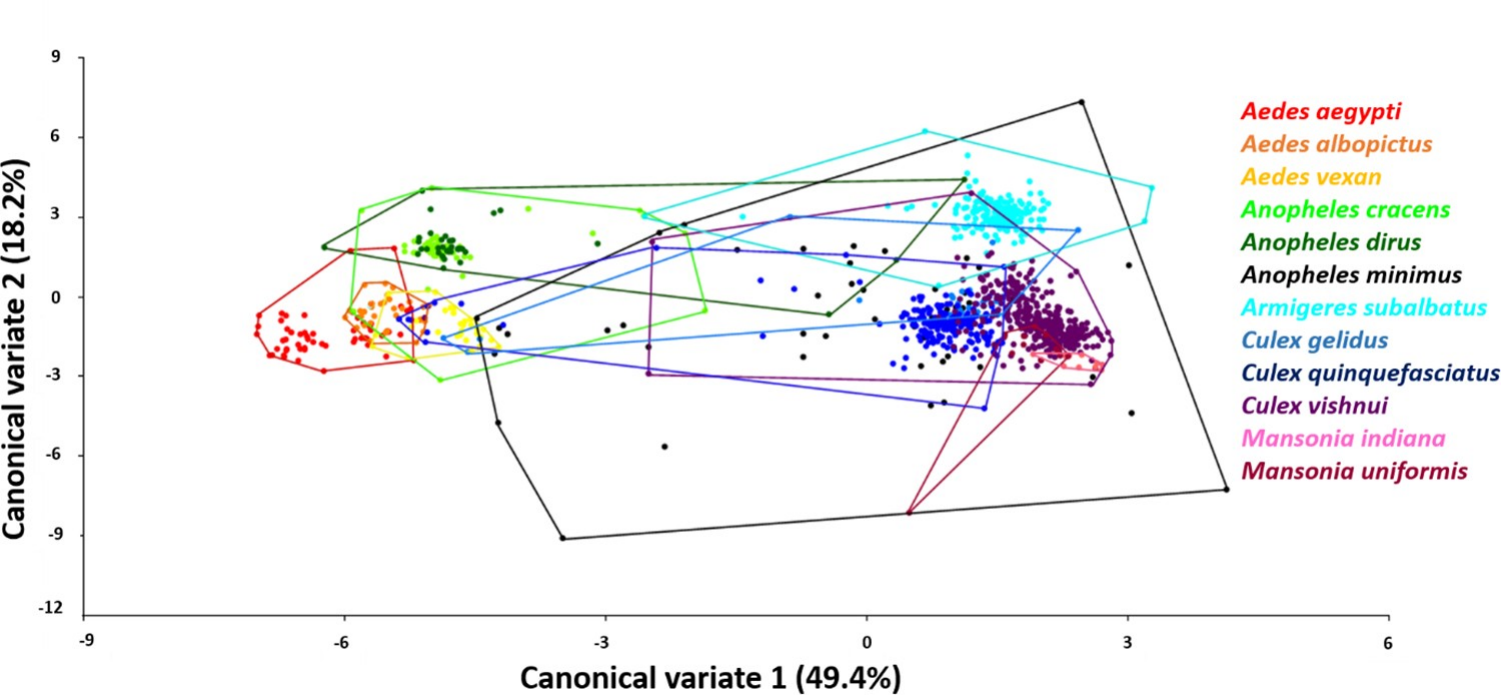
Scatter plot showing wing shape variation among 12 mosquito species. Wing shape variation is shown along the first CV1 and CV2 axes with 90% confidence ellipses.

The regression of Procrustes coordinates against CS revealed the allometry effect of wing size on wing shape (permutation test with 10,000 rounds in MorphoJ: 5.92%, *p* < 0.0001). Although small, the allometry effect was not removed from the analysis as we considered the allometric size variation of the species identification process [15]. CVA of wing shape among genera revealed five different clusters with morphological variation that were classified by color, including the mosquito genera *Aedes* (red), *Anopheles* (yellow), *Armigeres* (green), *Culex* (light blue), and *Mansonia* (purple; Fig 3). At the genus level, CVA revealed five canonical variates, among which the first two (Fig 3) explained 85.0% of the total variation (CV1 = 57.9%, CV2 = 27.1%). The scatter plot of CV1 and CV2 showed that *Aedes* and *Armigeres* specimens were separated into distinct groups, while *Mansonia* and *Culexs* specimens overlapped considerably with *Anopheles* specimens (Fig 3). At the species level (Fig 4), CVA explained 67.6% of the total variation (CV1 = 49.4%, CV2 = 18.2%), with the scatter plot revealing a morphometric difference between the Anophelinae and Culicinae subfamilies. In addition, overlapping was observed between all 12 mosquito species except *Ar*. *subalbatus*, which was clearly distinct.

The Mahalanobis distances obtained from pairwise comparisons between the 12 mosquito species ranged from 2.1351 (*An*. *dirus* and *An*. *cracens*) to 9.6419 (*Mn*. *indiana* and *Ae*. *aegypti*), with statistical analysis revealing significant differences (permutation test in MorphoJ: *p*< 0.0001; *p*< 0.01; and *p*< 0.05; S1 Table). Cross-validation (permutation test in MorphoJ) further showed that the percentage of specimens correctly classified by genus in the majority of comparisons ranged from 90.07% (*Culex*) to 97.48% (*Aedes*), with the exception of *Anopheles*, which had correct classification rates of less than 70.00% (Table 2).

**Table 2:** Percentage of specimens correctly classified by genus.

Genus	% correctly classified specimens	(No. correctly classified/total no. of specimens)
*Aedes*	97.48	(116/119)
*Anopheles*	64.54	(91/141)
*Armigeres*	96.15	(175/182)
*Culex*	90.07	(517/574)
*Mansonia*	91.67	(22/24)

The Procrustes coordinate distances of 12 mosquito species ranged from 0.0369 (*Mn*. *uniformis* and *Mn*. *indiana*) to 0.7542 (*Mn*. *indiana* and *Ae*. *albopictus*), with statistical analysis (permutation test in MorphoJ) indicating highly significant differences between most of these species (*p*< 0.0001; *p*< 0.01; and *p*< 0.05; S2 Table). The pairwise cross-validated reclassification test between mosquito species yielded a high percentage of correctly classified specimens in majority of the comparisons (80%-100%), except six pairwise comparisons, *Ae*. *albopictus* and *Ae*. *aegypti* (70%), *Ae*. *vexans* and *Ae*. *albopictus* (67%-76%), *An*. *minimus* and *An*. *cracens* (78%), *An*. *minimus* and *An*. *dirus* (71%), *An*. *minimus* and *Cx*. *gelidus* (78%), *An*. *minimus* and *Cx*. *quinquefasciatus* (67%), which had percentages of correct classification below 80%. When *Mn*. *indiana* was compared with *Mn*. *uniformis*, the lowest reclassification score was 0% in all 12 comparisons of the species (Table 3).

**Table 3:** Pairwise cross-validated species reclassification test.

		Group 2											
	Reclassification test	*Aedes aegypti*	*Aedes albopictus*	*Aedes vexans*	*Anopheles cracens*	*Anopheles dirus*	*Anopheles minimus*	*Armigeres subalbatus*	*Culex gelidus*	*Culex quinquefasciatus*	*Culex vishnui*	*Mansonia indiana*	*Mansonia uniformis*
**Group 1**	*Aedes aegypti*	x	89%	96%	100%	100%	98%	100%	100%	95%	100%	100%	100%
	*Aedes albopictus*	70%	X	76%	100%	100%	100%	100%	88%	94%	100%	100%	100%
	*Aedes vexans*	83%	67%	x	100%	100%	100%	100%	93%	93%	97%	100%	100%
	*Anopheles cracens*	100%	95%	98%	x	96%	96%	98%	96%	95%	98%	100%	100%
	*Anopheles dirus*	97%	94%	92%	86%	x	94%	97%	92%	92%	92%	100%	97%
	*Anopheles minimus*	82%	84%	90%	78%	71%	x	92%	78%	67%	80%	82%	80%
	*Armigeres subalbatus*	99%	99%	98%	99%	99%	99%	x	98%	99%	98%	100%	100%
	*Culex gelidus*	100%	95%	95%	95%	100%	92%	100%	x	95%	89%	100%	97%
	*Culex quinquefasciatus*	98%	99%	97%	99%	99%	97%	100%	94%	x	97%	100%	100%
	*Culex vishnui*	99%	99%	99%	99%	99%	98%	99%	95%	95%	x	99%	99%
	*Mansonia indiana*	100%	100%	100%	100%	100%	100%	100%	100%	100%	100%	x	0%
	*Mansonia uniformis*	100%	100%	100%	100%	100%	100%	100%	100%	100%	100%	0%	x

### Phenetic wing morphology relationships among species

**Fig 5:**
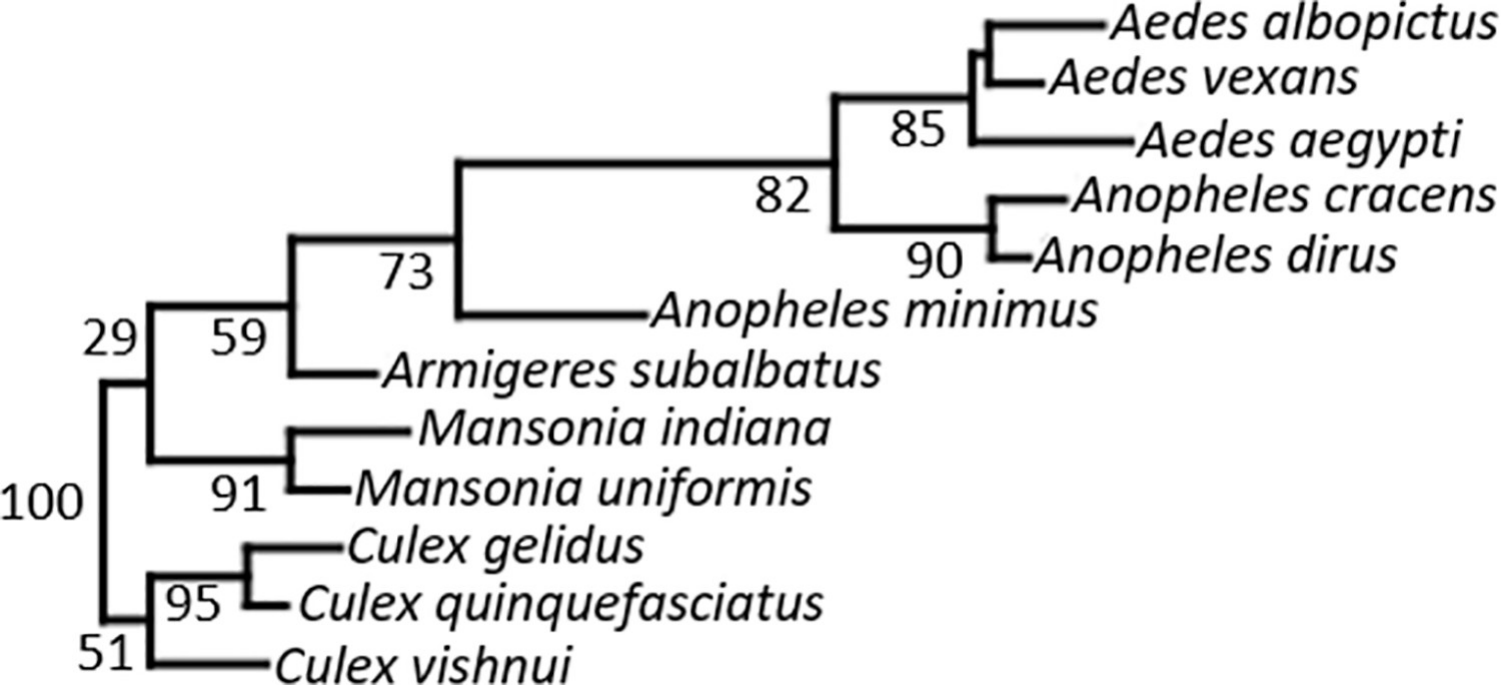
Neighbor-Joining tree showing phenetic wing morphology relationships among mosquito species. The tree was constructed based on the Mahalanobis distances between species.

The Neighbor-Joining tree showing the phenetic wing morphology relationships among the 12 mosquito species based on the Mahalanobis distances revealed two main clusters comprising the Culicinae and Anophelinae subfamilies. Three species from the genus *Culex* grouped together, with the highest levels of similarity between *Cx*. *gelidus* and *Cx*. *quinquefasciatus*. An identical pattern was observed for the genera *Mansonia* and *Aedes*; all the species clustered together in each genus branch. Regarding *Anopheles* species, they were placed into two distinct clusters (*An*. *minimus* and two sibling species; *An*. *cracens* and *An*. *dirus*) (Fig 5).

## Discussion

Morphological analysis using taxonomic keys is currently the standard method for identifying mosquito species; however, wing GM analysis also represents a reliable and inexpensive alternative that yields satisfactory results when discriminating between morphologically analogous species. In this study, we evaluated the ability of GM to correctly identify undamaged wing samples from 12 different mosquito species.

Although the number of laboratory strains was greater than the minimum number of samples per species required for precise genus and species differentiation using GM analysis, the number of some field strain mosquito species was beneath this threshold for clear species identification using GM. However, we found that the four important genera, *Aedes*, *Armigeres*, *Culex*, and *Mansonia*, could be correctly classified at the genus level using wing shape (90.07%-97.48%). In particular, the wing shape of *Aedes* and *Armigeres* species was clearly distinct from that of *Anopheles*, *Culex*, and *Mansonia* species. Conversely, the wing shape of *Anopheles* highly overlapped with that of *Culex* species (64.54% correctly classified). The broad overlapping indicated that these two sibling species members have a similar wing shape, which is morphologically identical (isomorphic) and has minimal morphological distinction. Therefore, correct identification among cryptic species based on morphological characteristics is difficult, which is consistent with previously reported findings [38, 39]. When cryptic diversity occurs in *Anopheles* and *Culex* (e.g., sibling, isomorphic, or cryptic species), molecular identification assays can help distinguish between samples [40–47]. In some studies, male and female genitalia have been used for reliable identification or for confirming identification of *Culex* species within the subgenus [48]. Our results revealed that the score of *Culex* species reclassification was higher than 80%, proving that these species can be identified through GM wing analysis. The reclassification score in the comparison of some pairs of species, including *Ae*. *albopictus* and *Ae*. *aegypti*, *Ae*. *vexans* and *Ae*. *albopictus*, *An*. *minimus* and *An*. *cracens*, *An*. *minimus* and *An*. *dirus*, *An*. *minimus* and *Cx*. *gelidus*, *An*. *minimus* and *Cx*. *quinquefasciatus*, and *Mn*. *uniformis* and *Mn*. *indiana* indicated low percentage of correct classification (0%-78%). Hence, it is recommended that GM be used in addition with traditional taxonomic identification keys or molecular tools for precise species identification. Species from the *Mansonia* genus were incorrectly identified with the lowest reclassification scores, which may explain that these two mosquitoes (*Mn*. *uniformis* and *Mn*. *indiana*) are members of the same subgenus *Mansonioides*, with similarities in wing shape structures. Therefore, the landmark-based analysis of wings can partially help to identify the genus level of *Mansonia* used in this study. However, morphological taxonomic keys needed for precise identification of *Mansonia* species require specific training and non-damaged wings for analysis [49].

We also produced a NJ tree showing the phenetic relationships in right-wing morphology based on the Mahalanobis distances between the 12 mosquito species examined in this study, which initially revealed two main clusters. The Anophelinae subfamilies were categorized into two clusters containing *An*. *minimus* complex (*An*. *minimus*) and *An*. *dirus* complex (*An*. *cracens* and *An*. *dirus*), which are the primary malaria vectors in Thailand. The *Anopheles* complex groups remain problematic because of overlapping morphological characters between sibling species. Therefore, wing GM analysis should be performed in combination with traditional morphological methods and molecular assays for accurate species identification within Anophelinae and Culicinae morphometric groups [44–48, 50].

Together, the results of this study demonstrate that landmark-based wing morphometry analysis could be an alternative tool for identifying mosquito species in Thailand, consistent with the findings of previous studies on three epidemiologically important genera (*Anopheles*, *Aedes*, and *Culex*) [15, 17, 23] and *Mansonia* [49]. However, this study utilized a limited number of *Ae*. *vexans*, *Mn*. *indiana*, and *Mn*. *uniformis* samples (30≤ N >10); therefore, further studies should be performed using more specimens from natural populations of these species to improve the reliability of wing shape analysis for species discrimination. Although landmark-based GM analysis can be time-consuming as a result of right-wing preparation and marking all the landmarks in duplicate samples, this method is less expensive and simpler than genetic sequencing, whilst still being reliable. The reliability of wing morphometric analysis depends on several factors: 1) consistent wing preparation to provide the most precise wing measurements; 2) using the same conditions and photographic equipment; and 3) wing landmark location by the same person and duplicate digitization to reduce data measurement errors. Thus, wing morphometric analysis requires less proficiency for an inexperienced person compared to standard taxonomic key identification. Moreover, the majority of female mosquitoes can be correctly identified even when using wing samples preserved in ethanol.

In conclusion, the findings of this study demonstrate that wing landmark-based GM analysis can be used to discriminate female mosquitoes at the genus and species levels. In particular, we found that this method is highly reliable when used for classification of *Aedes*, *Armigeres*, *Culex*, and *Mansonia* genera, but less reliable when used for discriminating *Anopheles* species, resulting in high percentage of correct classification for most mosquito species comparisons, except *Mansonia* species. However, the use of GM analysis could be an alternative technique, as it is easy to use, does not require proficient entomological skills, is a quick, practical, and simple technique, and has become particularly attractive for use in the field to facilitate the control of abundant vector species that are present in the area.

## Supporting information

S1 Table Wing shape variation among mosquito genera analyzed using CVA. (DOCX)

S2 Table CVA of wing shape variation among the 12 mosquito species analyzed. (DOCX)

## References

[1] Service MW. Biological control of mosquitoes- has it a future? Mosquito News. 1983;43(2):113–120.

[2] World Health Organization. Vector-Borne Diseases Factsheet. Geneva: WHO; 2020.

[3] Wilder-Smith A, Chen LH, Massad E, Wilson ME. Threat of dengue to blood safety in dengue-endemic countries. Emerg Infect Dis. 2009;15(1):8–11. doi: 10.3201/eid1501.071097 19116042 PMC2660677

[4] Rozendaal JA. Vector control: Methods for use by individuals and communities. Geneva: WHO; 1997.

[5] Herriman R. India: Dengue cases double, malaria cases down in 2015. Outbreak News Today. 2015 Dec 2 [Cited 2016 Sep 16 September]. Available from: http://outbreaknewstoday.com/india-dengue-cases-double-malaria-cases-down-in-2015-2015.

[6] Thongsripong P, Green A, Kittayapong P, Kapan D, Wilcox B, Bennett S. Mosquito vector diversity across habitats in central Thailand endemic for dengue and other arthropod-borne diseases. PLoS Negl Trop Dis. 2013;7(10):e2507. doi: 10.1371/journal.pntd.0002507 24205420 PMC3814347

[7] Chaiphongpachara T, Bunyuen P, Chansukh KK. Development of a more effective mosquito trapping box for vector control. Sci World J. 2018;2018:6241703. doi: 10.1155/2018/6241703 30154682 PMC6093027

[8] Chaiphongpachara T, Sumruayphol S. Species diversity and distribution of mosquito vectors in coastal habitats of Samut Songkhram Province, Thailand. Trop Biomed. 2017;34(3):524–532. 33592920

[9] Harbach RE. Culicipedia: Species-group, genus-group and family-group names in Culicidae (Diptera). Wallingford, Oxfordshire, UK: CABI, 2018.

[10] Gaffigan T, Pecor J. Collecting, rearing, mounting and shipping mosquitoes. Walter Reed Biosystematics Unit Division of Entomology, Walter Reed Army Institute of Research, 1997.

[11] Sabrosky CW. Mounting insects from alcohol. Bull Ent Soc Amer. 1966;12(3):349.

[12] Houba V. Handling, preservation, storage and transportation of biological materials. In Malaria: Principles and Practice of Malariology (Edited by Wernsdorfer W.H. and McGregor I.). Churchill Livingstone Publishers, Edinburgh, London, Melbourne, New York; 1988. pp. 1813–1818.

[13] Webster M, Sheets HD. A practical introduction to landmark- based geometric morphometrics. Paleontol Soc Papers. 2010;16:163–188.

[14] Dujardin JP. Modern morphometrics of medically important insects. In: Tibayrenc M, editor. Genetics and Evolution of Infectious Diseases. 2nd ed. Elsevier; 2017. pp. 473–501.

[15] Wilke ABB, Christe RdO, Multini LC, Vidal PO, Wilk-da-Silva R, de Carvalho GC, et al. Morphometric wing characters as a tool for mosquito identification. PLoS ONE. 2016;11(8):e0161643. doi: 10.1371/journal.pone.0161643 27551777 PMC4995034

[16] Chaiphongpachara T, Sriwichai P, Samung Y, Ruangsittichai J, Vargas REM, Cui L, et al. Geometric morphometrics approach towards discrimination of three member species of Maculatus group in Thailand. Acta Trop. 2019;192:66–74. doi: 10.1016/j.actatropica.2019.01.024 30710534 PMC7110943

[17] Prudhomme J, Velo E, Bino S, Kadriaj P, Mersini K, Gunay F, et al. Altitudinal variations in wing morphology of *Aedes albopictus* (Diptera, Culicidae) in Albania, the region where it was first recorded in Europe. Parasite. 2019;26:55. doi: 10.1051/parasite/2019053 31489838 PMC6729119

[18] Sontigun N, Sukontason KL, Zajac KB, Zehner R, Sukontason K, Wannasan A, et al. Wing morphometrics as a tool in species identification of forensically important blow flies of Thailand. Parasit Vectors. 2017;10:229. doi: 10.1186/s13071-017-2163-z 28486970 PMC5424331

[19] Changbunjong T, Sumruayphol S, Weluwanarak T, Ruangsittichai J. Landmark and outline-based geometric morphometrics analysis of three *Stomoxys* flies (Diptera: Muscidae). Folia Parasitologica. 2016;63:037. doi: 10.14411/fp.2016.037 27827335

[20] Ruangsittichai J, Apiwathnasorn C, Dujardin JP. Interspecific and sexual shape variation in the filariasis vectors *Mansonia dives* and *Ma*. *bonneae*. Infect Genet Evol. 2011;11(8):2089–2094. doi: 10.1016/j.meegid.2011.10.002 22020254

[21] Lorenz C, Marques TC, Sallum MAM, Suesdek L. Morphometrical diagnosis of the malaria vectors *Anopheles cruzii*, *An*. *homunculus* and *An*. *bellator*. Parasit Vectors. 2012;5:257. doi: 10.1186/1756-3305-5-257 23148743 PMC3514230

[22] Morales-Vargas REM, Phumala-Morales N, Tsunoda T, Apiwathnasorn C, Dujardin JP. The phenetic structure of *Aedes albopictus*. Infect Genet Evol. 2013;13:242–251. doi: 10.1016/j.meegid.2012.08.008 22985681

[23] Jaramillo- O N, Dujardin JP, Calle- Londoño D, Fonseca- González I. Geometric morphometrics for the taxonomy of 11 species of *Anopheles* (*Nyssorhynchus*) mosquitoes. Med Vet Entomol. 2015;29(1):26–36. doi: 10.1111/mve.12091 25393150

[24] Dujardin JP. Morphometrics applied to medical entomology. Infect Genet Evol. 2008;8(6):875–890. doi: 10.1016/j.meegid.2008.07.011 18832048

[25] Choochote W, Kanjanapothi D, Panthong A, Taesotikul T, Jitpakdi A, Chaithong U, et al. Larvicidal, adulticidal and repellent effects of *Kaempferia galanga*. Southeast Asian J Trop Med Public Health. 1999;30(3):470–476. 10774653

[26] Tuetun B, Choochote W, Pongpaibul Y, Junkum A, Kanjanapothi D, Chaithong U, et al. Field evaluation of G10, a celery (*Apium graveolens*)-based topical repellent, against mosquitoes (Diptera: Culicidae) in Chiang Mai province, northern Thailand. Parasitol Res. 2009;104:515–521. doi: 10.1007/s00436-008-1224-9 18853188

[27] Champakaew D, Junkum A, Chaithong U, Jitpakdi A, Riyong D, Wannasan A, et al. Assessment of *Angelica* *sinensis* (Oliv.) Diels as a repellent for personal protection against mosquitoes under laboratory and field conditions in northern Thailand. Parasit Vectors. 2016;9:373. doi: 10.1186/s13071-016-1650-y 27357395 PMC4928323

[28] Tanaka K, Mizusawa K, Saugstad ES. A revision of the adult and larval mosquitoes of Japan (including the Ryukyu Archipelago and the Ogasawara Islands) and Korea (Diptera: Culicidae). Contrib Amer Ent Inst. 1979;16:1–987.

[29] Rattanarithikul R, Panthusiri P. Illustrated keys to the medically important mosquitoes of Thailand. Southeast Asian J Trop Med Public Health. 1994;25:1–66. 7831585

[30] Rohlf FJ. TpsUtil32 v.1.78 software: tpsUtil (Version 1.78). SUNY Stony Brook: Stony Brook Morphometrics, 2019.

[31] Garzón MJ, Schweigmann N. Morphometric variation of the *Aedes albifasciatus* (Diptera: Culicidae) wings in three populations from different ecoregions of Argentina. J Med Entomol. 2018;55(6):1602–1606. doi: 10.1093/jme/tjy096 29939291

[32] Chaiphongpachara T, Laojun S. Variation over time in wing size and shape of the coastal malaria vector *Anopheles* (*Cellia*) *epiroticus* Linton and Harbach (Diptera: Culicidae) in Samut Songkhram, Thailand. J Adv Vet Anim Res. 2019;14:208–214. 10.5455/javar.2019.f334 PMC6702878 31453193

[33] Arnqvist G, Mårtensson T. Measurement error in geometric morphometrics: Empirical strategies to assess and reduce its impact on measures of shape. Acta Zool Academ Sci Hung. 1998;44(1):73–96.

[34] Rohlf FJ. TpsDig2 v.2.32 software: tpsDig (Version 2.32). SUNY Stony Brook: Stony Brook Morphometrics, 2018.

[35] Bookstein FL. Morphometric Tools for Landmark Data: Geometry and Biology. New York: Cambridge University Press; 1991.

[36] Haarlem C, Vos R. Inspecting morphological features of mosquito wings for identification with image recognition tools. BioRxiv [Preprint]. 2018 [Cited 2019 Feb 8 February]. Available from: https://www.biorxiv.org/content/10.1101/410449v1.full.

[37] Klingenberg CP. MorphoJ: an integrated software package for geometric morphometrics. Mol Ecol Resour. 2011;11(2):353–357. doi: 10.1111/j.1755-0998.2010.02924.x 21429143

[38] Bortel WV, Trung HD, Roelants P, Harbach RE, Backeljau T, Coosemans M. Molecular identification of *Anopheles minimus* s.l. beyond distinguishing the members of the species complex. Insect Mol Biol. 2000;9(3):335–340. doi: 10.1046/j.1365-2583.2000.00192.x 10886418

[39] Prakash A, Walton C, Bhattacharyya DR, Loughlin SO’, Mohapatra PK, Mahanta J. Molecular characterization and species identification of the *Anopheles dirus* and *An*. *minimus* complexes in north-east India using r-DNA ITS-2. Acta Trop. 2006;100(1–2):156–161. doi: 10.1016/j.actatropica.2006.09.009 17118324

[40] Taai K, Harbach RE. Systematics of the *Anopheles barbirostris* species complex (Diptera: Culicidae: Anophelinae) in Thailand. Zool J Linn Soc. 2015;174(2):244–264.

[41] Brosseau L, Udom C, Sukkanon C, Chareonviriyaphap T, Bangs MJ, Saeung A, et al. A multiplex PCR assay for the identification of five species of the *Anopheles barbirostris* complex in Thailand. Parasit Vectors. 2019;12(1):223. doi: 10.1186/s13071-019-3494-8 31088534 PMC6515612

[42] Suwannamit S. Identification of five sibling species of the *Anopheles barbirostris* complex (Diptera: Culicidae) in Thailand by a polymerase chain reaction assay. NUJST. 2021;29:1–8.

[43] Chan AHE, Chiang LP, Hapuarachchi C, Tan CH, Pang SC, Lee R. DNA barcoding: complementing morphological identification of mosquito species in Singapore. Parasit Vectors. 2014;7(1):569. 25498759 10.1186/s13071-014-0569-4 PMC4282734

[44] Afizah AN, Torno MM, Jannah JN, Azahari AH, Asuad KM, Nazni WA, et al. DNA barcoding complementing morphological taxonomic identification of mosquitoes in Peninsular Malaysia. Southeast Asian J Trop Med Public Health. 2019;50(1):36–46.

[45] Subbarao SK. Anopheline species complexes in South-East Asia. Technical Publication, SEARO No: 18. New Delhi: World Health Organization; 1998.

[46] Saeung A, Otsuka Y, Baimai V, Somboom P, Pitasawat B, Tuetun B, et al. Cytogenetic and molecular evidence for two species in the *Anopheles barbirostris* complex (Diptera: Culicidae) in Thailand. J Parasitol Res. 2007;101:1337–1344. 10.1007/s00436-007-0645-1 17659361

[47] Saeung A, Baimai V, Otsuka Y, Rattanarithikul R, Somboon P, Junkum A, et al. Molecular and cytogenetic evidence of three sibling species of the *Anopheles barbirostris* form A (Diptera: Culicidae) in Thailand. J Parasitol Res. 2008;102:499–507. 10.1007/s00436-007-0788-0 18038149

[48] Dehghan H, Sadraei J, Moosa-Kazemi SH. The morphological variations of *Culex pipiens* (Diptera: Culicidae) in central Iran. Asian Pac J Trop Med. 2011;4(3):215–219. doi: 10.1016/S1995-7645(11)60072-2 21771456

[49] Adeleke MA, Mafiana CF, Idowu AB, Adekunle MF, Dansu BM. Morphometric studies on *Culex quinquefasciatus* and *Mansonia africana* (Diptera: Culicidae) in Abeokuta, south-western Nigeria. Tanzan J Health Res. 2008;10(2):99–102. 18846788

[50] World Health Organization. Pictorial identification key of important disease vectors in the WHO South-East Asia Region. Regional Office for South-East Asia. 2020; https://apps.who.int/iris/handle/10665/332202. License: CC BY-NC-SA 3.0 IGO.

