# Supplementary material for: Geometric morphometric wing analysis as a tool to discriminate female mosquitoes from different suburban areas of Chiang Mai province, Thailand
Source: PLoS One. 2021 Nov 29;16(11):e0260333. doi: 10.1371/journal.pone.0260333 (PMC8629303; doi:10.1371/journal.pone.0260333)
Supplement: S1 Table — (DOCX) [file pone.0260333.s001.docx]

**S1** **Table. Wing shape variation among mosquito genera analyzed using CVA.**

|  | ***Aedes*** | ***Anopheles*** | ***Armigeres*** | ***Culex*** | ***Mansonia*** |
| --- | --- | --- | --- | --- | --- |
| ***Aedes*** | – | 0.2134*** | 0.5055*** | 0.4856*** | 0.7444*** |
| ***Anopheles*** | **5.1089***** | – | 0.3834*** | 0.3131*** | 0.5867*** |
| ***Armigeres*** | **7.6870***** | **5.8383***** | – | 0.2736*** | 0.6556*** |
| ***Culex*** | **6.1741***** | **5.1871***** | **3.9058***** | – | 0.3921*** |
| ***Mansonia*** | **6.9138***** | **6.1577***** | **5.8870***** | **2.9911***** | – |

Mahalanobis distances, bold type; Procrustes distances, standard type. Significant differences between genera (10,000 rounds of permutation analysis in Morpho J): ****p* < 0.0001; ***p* < 0.01; **p* < 0.05.
