# Supplementary material for: Geometric morphometric wing analysis as a tool to discriminate female mosquitoes from different suburban areas of Chiang Mai province, Thailand
Source: PLoS One. 2021 Nov 29;16(11):e0260333. doi: 10.1371/journal.pone.0260333 (PMC8629303; doi:10.1371/journal.pone.0260333)
Supplement: S2 Table — (DOCX) [file pone.0260333.s002.docx]

**S2** **Table**. **CVA of wing shape variation among the 12 mosquito species analyzed.**

| Species code | *Ae. aegypti* | *Ae. albopictus* | *Ae. vexans* | *An. cracens* | *An. dirus* | *An. minimus* | *Ar. subalbatus* | *Cx. gelidus* | *Cx. quinquefasciatus* | *Cx. vishnui* | *Mn. indiana* | *Mn. uniformis* |
| --- | --- | --- | --- | --- | --- | --- | --- | --- | --- | --- | --- | --- |
| *Ae. aegypti* | - | 0.1317*** | 0.1438*** | 0.1713*** | 0.1711*** | 0.4549*** | 0.5115*** | 0.4339*** | 0.4461*** | 0.5833*** | 0.7532*** | 0.7505*** |
| *Ae. albopictus* | **4.5066**  ******* | - | 0.0544*** | 0.0978*** | 0.1268*** | 0.4481*** | 0.5148*** | 0.4359*** | 0.4507*** | 0.5820*** | 0.7542*** | 0.7526*** |
| *Ae. vexans* | **5.0592**  ******* | **3.5554**  ******* | - | 0.1074*** | 0.1251*** | 0.4326*** | 0.5031*** | 0.4233*** | 0.4368*** | 0.5633*** | 0.7358*** | 0.7339*** |
| *An. cracens* | **5.8731**  ******* | **4.7068**  ******* | **5.6654**  ******* | - | 0.0549* | 0.3860*** | 0.4770*** | 0.3979*** | 0.4140*** | 0.5278*** | 0.7034*** | 0.7003*** |
| *An. dirus* | **6.3444**  ******* | **5.5690**  ******* | **6.1496**  ******* | **2.1351**  ****** | - | 0.3617*** | 0.4573*** | 0.3776*** | 0.3933*** | 0.5078*** | 0.6846*** | 0.6804*** |
| *An. minimus* | **9.3896**  ******* | **8.5057**  ******* | **9.3606**  ******* | **7.6507**  ******* | **7.9294**  ******* | - | 0.3203*** | 0.2545*** | 0.2739*** | 0.2195*** | 0.4288*** | 0.4211*** |
| *Ar. subalbatus* | **9.1154**  ******* | **8.2692**  ******* | **8.2592**  ******* | **7.0061**  ******* | **6.9817**  ******* | **7.3647**  ******* | - | 0.1082*** | 0.0944*** | 0.4268*** | 0.6608*** | 0.6516*** |
| *Cx. gelidus* | **7.7090**  ******* | **6.9055**  ******* | **6.6558**  ******* | **6.7051**  ******* | **6.6884**  ******* | **6.2297**  ******* | **3.7132**  ******* | - | 0.0429* | 0.3798*** | 0.6154*** | 0.6079*** |
| *Cx. quinquefasciatus* | **7.7340**  ******* | **7.2097**  ******* | **6.9751**  ******* | **6.8231**  ******* | **6.9723**  ******* | **6.0255**  ******* | **4.6633**  ******* | **2.3007**  ******* | - | 0.4012*** | 0.6357*** | 0.6271*** |
| *Cx. vishnui* | **8.6541**  ******* | **7.9185**  ******* | **7.4862**  ******* | **7.4760**  ******* | **7.4351**  ******* | **6.6387**  ******* | **4.2260**  ******* | **2.6273**  ******* | **3.3413**  ******* | - | 0.2424** | 0.2355** |
| *Mn. indiana* | **9.6419**  ******* | **9.1218**  ******* | **8.5245**  ******* | **8.9220**  ******* | **8.9195**  ******* | **7.5255**  ****** | **6.4534**  ******* | **4.5941**  ******* | **4.9202**  ******* | **3.2461**  ****** | - | 0.0369** |
| *Mn. uniformis* | **9.0707**  ******* | **8.4904**  ******* | **8.2734**  ******* | **8.2352**  ******* | **8.2273**  ******* | **6.4748**  ****** | **6.1813**  ******* | **4.2865**  ******* | **4.5106**  ******* | **2.9770**  ****** | **2.1742**  ****** | - |

Mahalanobis distances, bold type; Procrustes distances, standard type. Significant differences between species (10,000 rounds of permutation analysis in Morpho J): ****p* < 0.0001; ***p* < 0.01; **p* < 0.05).
